# Hybrid Decoding with Co-Occurrence Awareness for Fine-Grained Food Image Segmentation

**DOI:** 10.3390/foods15030534

**Published:** 2026-02-03

**Authors:** Shenglong Wang, Guorui Sheng

**Affiliations:** School of Computer Science and Artificial Intelligence, Ludong University, Yantai 264025, China; wangshenglong@m.ldu.edu.cn

**Keywords:** food computing, deep learning, food image segmentation, food co-occurrence

## Abstract

Fine-grained food image segmentation is essential for accurate dietary assessment and nutritional analysis, yet remains highly challenging due to ambiguous boundaries, inter-class similarity, and dense layouts of meals containing many different ingredients in real-world settings. Existing methods based solely on CNNs, Transformers, or Mamba architectures often fail to simultaneously preserve fine-grained local details and capture contextual dependencies over long distances. To address these limitations, we propose HDF (Hybrid Decoder for Food Image Segmentation), a novel decoding framework built upon the MambaVision backbone. Our approach first employs a convolution-based feature pyramid network (FPN) to extract multi-stage features from the encoder. These features are then thoroughly fused across scales using a Cross-Layer Mamba module that models inter-level dependencies with linear complexity. Subsequently, an Attention Refinement module integrates global semantic context through spatial–channel reweighting. Finally, a Food Co-occurrence Module explicitly enhances food-specific semantics by learning dynamic co-occurrence patterns among categories, improving segmentation of visually similar or frequently co-occurring ingredients. Evaluated on two widely used, high-quality benchmarks, FoodSeg103 and UEC-FoodPIX Complete, which are standard datasets for fine-grained food segmentation, HDF achieves a 52.25% mean Intersection-over-Union (mIoU) on FoodSeg103 and a 76.16% mIoU on UEC-FoodPIX Complete, outperforming current state-of-the-art methods by a clear margin. These results demonstrate that HDF’s hybrid design and explicit co-occurrence awareness effectively address key challenges in food image segmentation, providing a robust foundation for practical applications in dietary logging, nutritional estimation, and food safety inspection.

## 1. Introduction

In recent years, rapid economic development and globalization have profoundly diversified dietary patterns worldwide, enabling individuals to access a wide variety of foods across cultures and seasons [1]. While this diversification has enriched food availability, it has also contributed to a global rise in nutrition-related health issues, including obesity, malnutrition, and chronic metabolic diseases. These challenges are largely driven by imbalanced food intake and insufficient nutritional awareness among the general public [2,3]. As a result, there is an urgent demand for intelligent and accessible technologies that can assist individuals in monitoring daily food consumption and making informed dietary decisions, thereby supporting healthier agri-food systems and improved public health outcomes [4].

With the rapid advancement of artificial intelligence (AI), food computing has emerged as a critical research direction at the intersection of computer vision and agri-food systems [5]. Core perception tasks—including food recognition, detection, and segmentation—form the foundation of food computing pipelines and play an essential role in downstream applications such as dietary assessment, food safety inspection, and nutritional analysis [6]. Among these tasks, food image segmentation is particularly important, as it enables pixel-level delineation of individual food items in complex meal images. Accurate segmentation not only identifies food categories and precise contours but also supports the estimation of key physical attributes such as volume and mass when combined with RGB-D or multimodal sensing data [7,8]. These capabilities are crucial for precise nutritional assessment and food-safety-related applications [9,10].

Despite its importance, food image segmentation remains a highly challenging problem due to the intrinsic complexity of food imagery [11]. First, strong inter-class similarity is common in food scenes; for example, cooked tomatoes and red bell peppers exhibit nearly indistinguishable color and texture, while different types of rice or pasta differ only subtly in shape. Second, intra-class variation is extreme, as the same ingredient may appear mashed, fried, roasted, or mixed with sauces, leading to drastically different visual appearances. Third, real-world meals often involve dense multi-ingredient arrangements, severe occlusions, ambiguous boundaries caused by sauces or steam, and highly unstructured plating styles. These factors collectively render fine-grained food segmentation substantially more difficult than generic semantic segmentation tasks.

Early semantic segmentation methods were primarily based on fully convolutional networks (FCNs), such as U-Net [12], PSPNet [13], and DeepLabV3+ [14], which established strong baselines through hierarchical convolutional feature extraction. In the food computing domain, classical convolutional neural network (CNN) architectures were widely adopted in early studies [15,16]. Although effective in extracting local texture features, these CNN-based approaches are inherently limited by their local receptive fields, restricting their ability to model long-range semantic dependencies. This limitation is particularly problematic for food image segmentation, where visually similar ingredients may appear in distant regions of an image or where occluded boundaries require global contextual reasoning.

To overcome these limitations, the introduction of Vision Transformers (ViTs) [17] and self-attention mechanisms has led to a new generation of segmentation models, including SegFormer [18], Swin Transformer [19], and Mask2Former [20]. These models excel at capturing global contextual information and have consistently outperformed CNN-based methods on various segmentation benchmarks. The global modeling capability of ViTs has also proven beneficial for food image understanding. Recent studies have fine-tuned the Segment Anything Model (SAM) [21] on food datasets [22,23], while Min et al. [24] proposed PRENet, which combines multi-stage local feature learning with self-attention to enhance fine-grained food discrimination. However, the quadratic computational complexity of self-attention introduces substantial memory overhead and slow inference speed when processing high-resolution food images, limiting real-time deployment on resource-constrained devices such as smartphones.

Hybrid CNN–Transformer architectures, such as NextViT [25] and FasterViT [26], attempt to balance local feature extraction and global context modeling for general vision tasks. Nevertheless, these models are not inherently designed to address the unique visual characteristics of food imagery and dense multi-ingredient layouts. Even recent adaptations of hybrid architectures to food recognition tasks [27] exhibit limitations in long-range context modeling. More recently, Mamba, a selective State Space Model (SSM) architecture [28], has introduced a new paradigm for efficient long-sequence modeling. Representative models such as Vim [29] and VMamba [30] demonstrate that global context aggregation can be achieved with linear computational complexity. However, approaches relying solely on Mamba often sacrifice fine-grained local detail, which is essential for accurate food image segmentation.

Despite the significant progress made by the aforementioned approaches in either general or food segmentation tasks, they share a common limitation: the inability to jointly optimize local detail fidelity, global contextual modeling, and semantic priors that are specific to the food domain within a unified framework. For instance, CNNs emphasize local features but lack long-range awareness; Transformers capture global context yet often overlook boundary precision; and even current hybrid architectures, while integrating multiple paradigms, fail to explicitly model the co-occurrence characteristics of visually similar ingredients that are inherent in real-world meals. To bridge this gap, MambaVision [31] proposes a unified hybrid backbone that integrates CNNs, Transformers, and Mamba within a single architecture. When combined with the standard UperNet decoder [32], MambaVision achieves strong performance on general segmentation benchmarks. Nevertheless, its fixed-topology feature fusion strategy is insufficient for the nuanced demands of food image segmentation. In particular, it lacks the adaptability required to resolve fine-grained boundaries between visually similar ingredients and to model the dynamic, spatially structured co-occurrence patterns inherent in food scenes. Although recent studies have incorporated food co-occurrence priors via constraints in the loss function [33], such approaches remain limited by their static nature and lack of explicit spatial awareness.

These challenges collectively limit the reliability of current food perception pipelines in practical agri-food and nutrition-related systems. To address these limitations, we propose HDF (Hybrid Decoder for Food Image Segmentation), a novel decoder architecture built upon the MambaVision backbone. Compared to existing approaches, HDF introduces a decoding mechanism that effectively unifies local, global, and domain-aware representations, thereby alleviating ambiguity in distinguishing co-occurring or visually similar food items and enhancing robustness in complex, real-world dietary scenarios.The core innovation of HDF lies in the synergistic integration of three complementary representational streams: the fine-grained local detail perception of CNNs, the global semantic reasoning capability of Transformers, and the efficient long-range dependency modeling of Mamba. This tripartite design enables the decoder to preserve texture fidelity while capturing contextual relationships across complex food scenes, while also maintaining computational efficiency for high-resolution images.

Specifically, HDF adopts a progressive five-stage decoding architecture: (i) a hierarchical feature pyramid for multi-scale feature fusion; (ii) a Cross-Layer Mamba module that models inter-layer dependencies with linear complexity, alleviating information bottlenecks in conventional feature pyramid networks; (iii) a Multi-Scale Enhancement module that expands receptive fields through parallel convolutions; (iv) an Attention Refinement block that sharpens ambiguous boundaries via spatial–channel reweighting; and (v) a spatially aware Food Co-occurrence Module (FCM) that learns dynamic, pixel-wise co-occurrence patterns in a data-driven manner, moving beyond static, hand-crafted priors.

## 2. Materials and Methods

### 2.1. Datasets

In this study, two publicly available food image segmentation datasets were employed to comprehensively evaluate the effectiveness and generalization capability of the proposed method across diverse food scenes.

FoodSeg103 [34] is a large-scale, fine-grained food image segmentation dataset designed to support detailed food understanding in nutrition- and health-related applications. The dataset consists of 7118 images of Western-style meals, covering 103 ingredient categories. Each image is annotated with pixel-level segmentation masks, containing an average of six ingredient instances per image and approximately 42,000 annotated masks in total.

FoodSeg103 was constructed based on the Recipe1M dataset [35] and refined through rigorous data filtering and multiple rounds of manual annotation and correction. This process ensures high-quality semantic labels and accurate boundary delineation, which are essential for reliable evaluation of fine-grained food segmentation methods. As a result, FoodSeg103 has been widely adopted as a benchmark for research on food image segmentation and downstream tasks such as nutritional estimation and dietary analysis.

**Figure 1:**
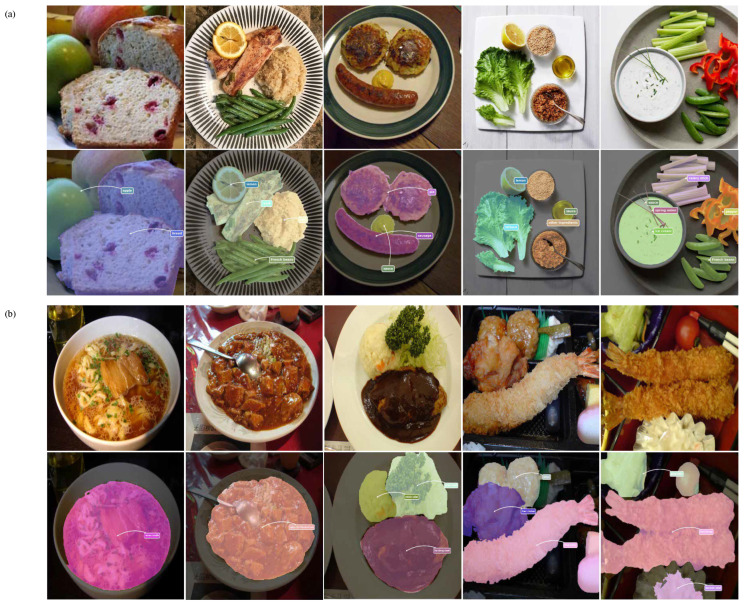
Example annotations from two food segmentation datasets: (**a**) FoodSeg103 and (**b**) UEC-FoodPIX Complete. The top row shows the original food images, while the bottom row presents the corresponding ground-truth segmentation masks. FoodSeg103 focuses on fine-grained ingredient-level labeling with challenging co-occurring items, whereas UEC-FoodPIX Complete emphasizes pixel-level delineation of entire dishes, reflecting distinct annotation granularities and application scopes.

As illustrated in Figure 1, the dataset provides precise pixel-level annotations for individual food components, including bread, beef, tomato, lemon, and sauce, reflecting the complex visual compositions and ingredient co-occurrence patterns commonly encountered in real-world meal images. These characteristics make FoodSeg103 particularly suitable for assessing segmentation performance under challenging food scene conditions involving inter-class similarity, occlusion, and ambiguous boundaries.

In addition to FoodSeg103, we further evaluated the proposed method on the UEC-FoodPIX Complete dataset [36], a widely used benchmark for food image segmentation that focuses on complex, real-world meal scenes. UEC-FoodPIX Complete is an extension of the UEC-FoodPIX dataset and provides pixel-level annotations for food items commonly encountered in Japanese cuisine, making it complementary to FoodSeg103 in terms of cultural context and food composition diversity.

The dataset contains 10,000 food images covering 103 food categories, with each image annotated at the pixel level to delineate individual food regions. Compared with Western-style meal datasets, UEC-FoodPIX Complete features distinctive visual characteristics, including frequent use of mixed dishes, overlapping ingredients, and visually similar food components presented within a single plate. These characteristics pose additional challenges for fine-grained food image segmentation, particularly in distinguishing adjacent food regions with subtle texture and color differences.

UEC-FoodPIX Complete has been extensively adopted in prior studies for evaluating food segmentation and recognition algorithms, owing to its realistic presentation styles and high-quality annotations. By incorporating both FoodSeg103 and UEC-FoodPIX Complete in our evaluation, we aim to assess the robustness and generalization capability of the proposed method across culturally diverse food scenes and varying ingredient co-occurrence patterns. Representative examples from the dataset are shown in Figure 1, illustrating the complexity of food arrangements and the necessity of context-aware segmentation strategies.

### 2.2. Method Overview

Food image segmentation in real-world scenarios requires a careful balance between fine-grained local detail preservation and global contextual understanding. As discussed in the Introduction, food images are characterized by complex textures, ambiguous boundaries, multi-scale object distributions, and frequent co-occurrence of visually similar ingredients. These challenges limit the effectiveness of conventional single-paradigm architectures and highlight the need for a more flexible and context-aware segmentation framework.

To address these challenges, we propose HDF (Hybrid Decoder for Food Image Segmentation), a novel decoder architecture built upon the MambaVision backbone. The central idea of HDF is to synergistically integrate complementary representational mechanisms to capture diverse visual cues present in food scenes. Specifically, HDF combines the fine-grained local feature extraction capability of convolutional neural networks (CNNs), the global semantic reasoning power of Transformer-based self-attention, and the efficient long-range dependency modeling of the Mamba State Space Model. This hybrid design enables robust segmentation performance while maintaining computational efficiency for high-resolution food images.

The proposed HDF framework follows a modular decoding strategy that progressively refines feature representations across multiple scales. It incorporates an enhanced feature pyramid network for multi-scale feature fusion, a Cross-Layer Mamba module for modeling inter-layer dependencies, Multi-Scale Enhancement blocks to expand receptive fields, and Attention Refinement mechanisms to sharpen ambiguous boundaries. In addition, a spatially aware Food Co-occurrence Module is introduced to explicitly model semantic relationships among food categories, leveraging domain-specific knowledge about ingredient combinations commonly observed in real-world meals.

**Figure 2:**
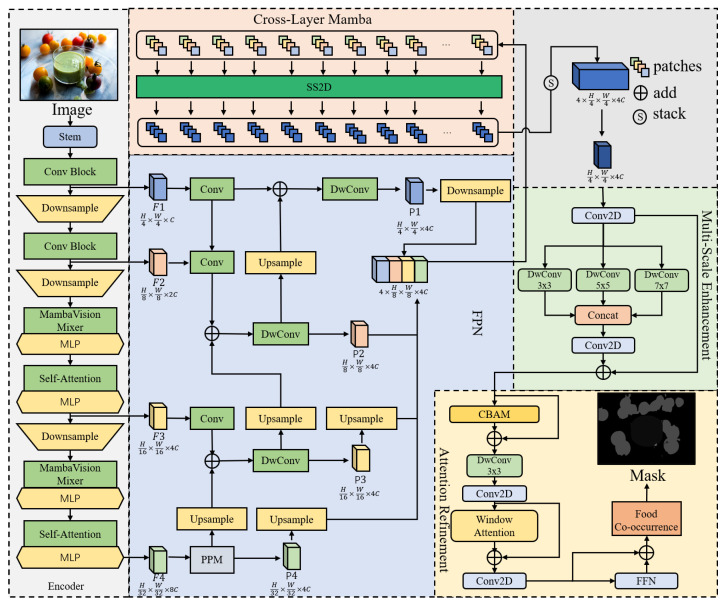
Overall architecture of the proposed HDF decoder. It takes multi-scale features F1, F2, F3, F4 from the MambaVision encoder and refines them through five key components, Enhanced FPN, Cross-Layer Mamba, Multi-Scale Enhancement, Attention Refinement, and the Food Co-occurrence Module, ultimately producing a high-quality segmentation mask.

An overview of the proposed architecture is illustrated in Figure 2. The following subsections describe each component of HDF in detail, including their design motivations, architectural configurations, and roles in addressing the challenges of fine-grained food image segmentation.

#### 2.2.1. Hybrid Encoder for Food Image Representation

To effectively capture both fine-grained local details and long-range contextual dependencies in complex food images, we adopt the MambaVision backbone as our hybrid encoder. This architecture synergistically combines Convolutional Neural Networks (CNNs) for local feature extraction with Mamba and Transformer blocks for global context modeling, processing the input through four hierarchical stages.

Given an input image $I\in R^{H\times W\times 3}$, a stem module implemented as two consecutive 3 × 3 convolutional layers with stride 2 performs initial downsampling and projects the features into a C-dimensional embedding space (detailed formulations are provided in Appendix A).

**Figure A1:**
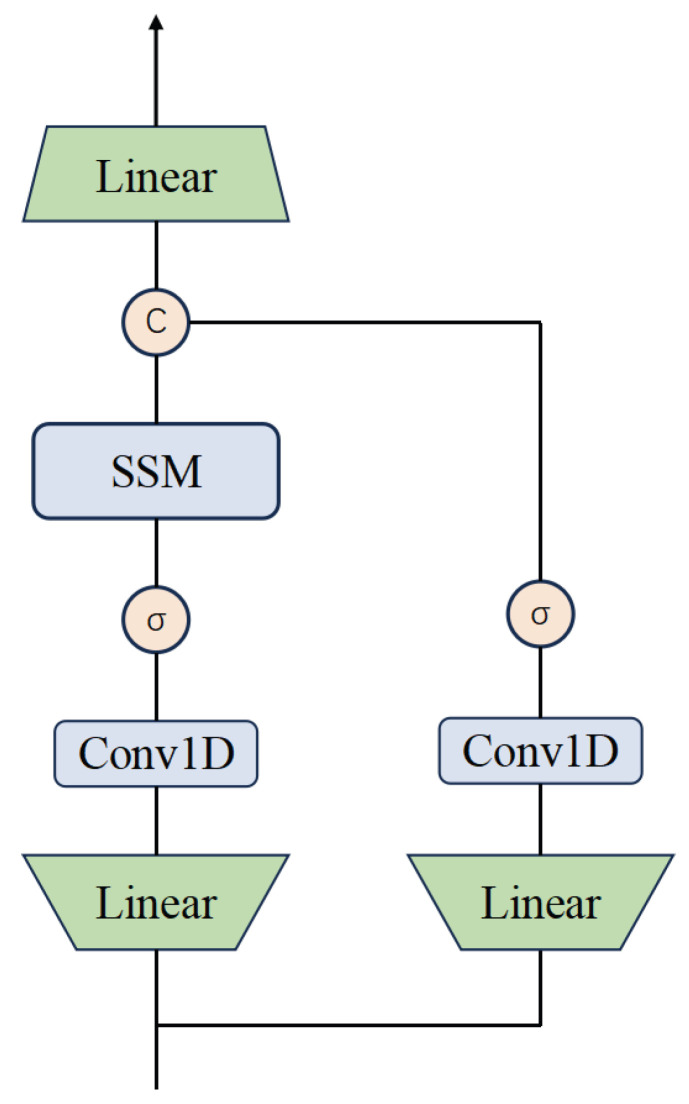
Structure of the MambaVision mixer. The input is processed by an SSM module, then split into two parallel branches. One branch applies a 1D convolution and linear projection, while the other uses a SiLU activation followed by a 1D convolution and linear projection. The outputs are concatenated for final feature representation.

The first two stages (Stage 0 and Stage 1) employ a pure CNN architecture with residual blocks to rapidly extract high-resolution local features. The latter two stages (Stage 2 and Stage 3) adopt the MambaVisionMixer design, the specific structural diagram is shown in Figure A1, which alternately stacks Mamba and Transformer layers. The Mamba block, a vision-adapted State Space Model (SSM), replaces causal convolution with standard convolution and incorporates a symmetric branch to facilitate bidirectional context modeling, addressing the sequential limitation of the original SSM. Its selectivity mechanism dynamically adjusts parameters based on the input, enabling global dependency capture with linear complexity O(T·C). The Transformer block, in turn, employs conventional multi-head self-attention to model global semantic relationships.

This hybrid design balances efficiency and expressiveness. It outputs four multi-scale feature maps $\left\{F_{1},F_{2},F_{3},F_{4}\right\}$ with channel dimensions {C,2C,4C,8C}, which collectively encode a rich hierarchy of information from low-level textures to high-level semantics. Specifically, Stage 2 produces $F_{3}\in R^{H/16\times W/16\times 4C}$, and Stage 3 produces $F_{4}\in R^{H/32\times W/32\times 8C}$.

#### 2.2.2. Hybrid Decoder with Co-Occurrence Awareness

To progressively refine multi-scale representations and address the complex spatial layouts commonly observed in food images, the proposed HDF decoder receives multi-scale features $\left\{F_{1},F_{2},F_{3},F_{4}\right\}$ from the encoder and fuses them through five core modules, as illustrated in Figure 2. First, it employs depthwise separable convolution (DwConv) to replace the standard convolution in traditional FPNs. The depthwise separable convolution decomposes the standard convolution into a depthwise convolution and a pointwise convolution, reducing the parameter count from $k^{2}\cdot C_{in}\cdot C_{out}$ to $k^{2}\cdot C_{in}+C_{in}\cdot C_{out}$. The features at all four scales are first unified to a base dimension *D* via 1×1 convolutions, and then fused using a top-down pathway:

(1) $$F_{i}^{\prime }=\mathrm{BN}\left({\mathrm{Conv}}_{1\times 1}^{C_{i}\to D}\left(F_{i}\right)\right),\;\forall i\in \left\{1,2,3,4\right\}$$

(2) $$P_{4}=F_{4}^{\prime }$$

(3) $$P_{i}={\mathrm{DwConv}}_{3\times 3}\left(F_{i}^{\prime }+{\mathrm{Upsample}}_{2\times }\left(P_{i+1}\right)\right),\;\forall i\in \left\{3,2,1\right\}$$

**Figure 3:**
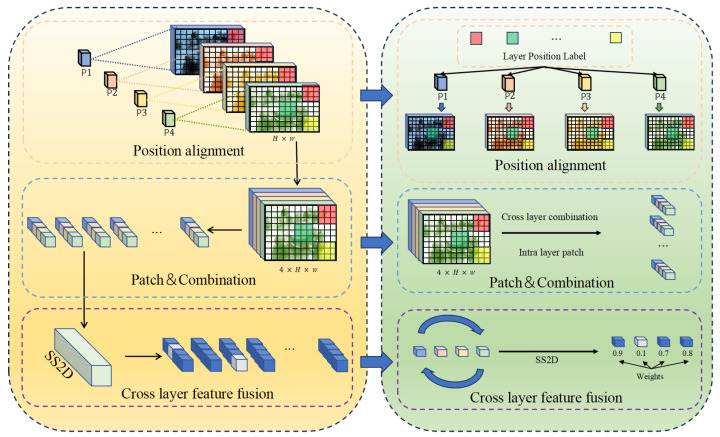
Illustration of the Cross-Layer Mamba module. It takes four multi-scale feature maps (P1, P2, P3, P4) from the encoder, aligns their positions to a common resolution, and then processes them through a sequence of operations: (1) position alignment with learnable layer embeddings; (2) Patch and Combination, where features from different layers are combined into a tensor; and (3) cross-layer feature fusion via the SS2D mechanism, which assigns adaptive weights to different levels at each spatial location for efficient inter-layer dependency modeling.

Next, as shown in the Figure 3, the **Cross-Layer Mamba** module leverages selective State Space Models to achieve cross-layer feature fusion while preserving spatial structure. Inspired by the 2D-selective-scan (SS2D) technique proposed in VMamba, we adapt this approach for modeling dependencies between different hierarchical levels in FPN structures. Instead of applying SS2D to individual feature maps, we employ it to capture relationships across feature levels at each spatial location. Given the four feature maps $F=\{F_{1},F_{2},F_{3},F_{4}\}$ from the encoder, we first upsample them to a common intermediate resolution of H/2×W/2 and unify their representation by adding learnable level embeddings:

(4) $${\tilde{F}}_{l}=\mathrm{Interpolate}\left(F_{l},\left(H,W\right)\right)+E_{l}$$

where $E_{l}$ denotes the embedding for the *l*-th level. To enable efficient long-range interaction across layers, the unified feature tensor is divided into non-overlapping patches of size 32×32. Within each patch, for every spatial position (i,j), we construct a cross-level feature sequence:

(5) $$x_{i,j}=\left[{\tilde{F}}_{1}^{\left(i,j\right)},{\tilde{F}}_{2}^{\left(i,j\right)},{\tilde{F}}_{3}^{\left(i,j\right)},{\tilde{F}}_{4}^{\left(i,j\right)}\right]$$

These sequences are then processed via the selective state space equations:

(6) $$h_{t+1}={\bar{A}}_{t}h_{t}+{\bar{B}}_{t}x_{t},\;y_{t}=C_{t}h_{t}+Dx_{t}$$

where ${\bar{A}}_{t}=\exp\left(\Delta_{t}⊙A\right)$, ${\bar{B}}_{t}=\Delta_{t}⊙B_{t}$, and $\Delta_{t}=\mathrm{softplus}\left(\mathrm{Linear}\left(x_{t}\right)\right)$ serves as an adaptive time step that selectively propagates information across levels. After Mamba-based propagation, all patches are reassembled into the original spatial layout. Finally, a lightweight global attention mechanism is applied to adaptively fuse the features from the four levels, yielding a single enhanced feature map $F_{\mathrm{fused}}$ that effectively integrates multi-scale contextual information.

The **Multi-Scale Enhancement** module expands the receptive field through a parallel multi-branch structure. Food images contain objects at multiple scales, ranging from entire dishes to fine ingredients. This module first reduces the dimensionality of the input feature map $F_{fused}$ by half via a 1×1 convolution. It then employs three parallel branches using depthwise separable convolutions of different kernel sizes: 3×3 (for local features), 5×5 (for medium receptive fields), and 3×3 dilated convolution (with dilation rate = 2, yielding an effective receptive field of 7×7). The outputs of the three branches are concatenated and then fused via a 1×1 convolution:

(7) $$F_{r}=\mathrm{ReLU}\left(\mathrm{BN}\left({\mathrm{Conv}}_{1\times 1}^{C\to C/2}\left(F_{fused}\right)\right)\right)$$

(8) $$B_{i}={\mathrm{DwConv}}_{k_{i}}\left(F_{r}\right),\;i\in \left\{1,2,3\right\}$$

(9) $$F_{MSEnhanced}=\mathrm{ReLU}\left(\mathrm{BN}\left({\mathrm{Conv}}_{1\times 1}\left(\mathrm{Concat}\left[B_{1};B_{2};B_{3}\right]\right)\right)\right)$$

The **Attention Refinement** module combines channel attention and spatial attention to mitigate the inter-class similarity problem in food segmentation. This module first enhances the feature representation via **CBAM** (Channel-and-Spatial-Attention Module). To reduce computational complexity, the features are downsampled to dimension D/2, fed into **WindowAttention** for intra-window attention modeling (with window size 8×8), and then upsampled back to dimension *D*. Finally, the refined features are obtained through a lightweight feed-forward network (**FFN**) and multi-layer residual connections, yielding the final refined feature map $F_{AR}$.

**Figure 4:**
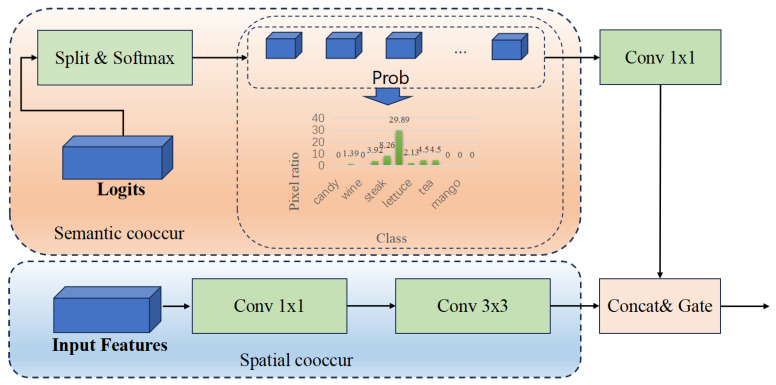
Architecture of the proposed Food Co-occurrence Module (FCM). The module consists of two parallel paths: Semantic Co-occurrence (**top**) and Spatial Co-occurrence (**bottom**).

We introduce the **Food Co-occurrence Module** (FCM), a dual-path architecture designed to model both Semantic and Spatial Co-Occurrence patterns in food images. As illustrated in Figure 4, the module processes two inputs: the feature map $X\in R^{B\times C\times H\times W}$ and the classification logits $L\in R^{B\times N\times H\times W}$, where *B*, *C*, *H*, *W*, and *N* denote batch size, channel dimension, height, width, and total number of classes (including background), respectively.

**Semantic Co-occurrence Path**: The semantic path begins by extracting the probability distribution for all food classes (excluding background) from the logits:

(10) $$P_{\mathrm{food}}=\mathrm{Softmax}\left(L_{\left[:,1:N\right]}\right)\in R^{B\times (N-1)\times H\times W}$$

A 1×1 convolution projects these probabilities into a low-dimensional semantic space, generating $F_{\mathrm{sem}}$. A subsequent lightweight block, comprising a 1×1 convolution, batch normalization, and ReLU activation, models global semantic interactions, producing the enhanced semantic feature $F_{\mathrm{sem}}^{*}$.

**Spatial Co-occurrence Path**: The spatial path operates directly on the input feature *X*. It first applies a 1×1 convolution for channel reduction, followed by a depthwise 3×3 convolution (implemented as a grouped convolution with a group count equal to the reduced channel dimension) to aggregate local spatial context. This yields the enhanced spatial feature $F_{\mathrm{spat}}^{*}$.

**Feature Fusion and Adaptive Gating**: The enhanced semantic and spatial features, $F_{\mathrm{sem}}^{*}$ and $F_{\mathrm{spat}}^{*}$, are concatenated and fused through a two-layer 1×1 convolution network to produce the co-occurrence feature $F_{\mathrm{cooccur}}$.

To enable spatially adaptive fusion, we introduce a gating mechanism. The original feature *X* and the co-occurrence feature $F_{\mathrm{cooccur}}$ are concatenated and processed by a small two-layer convolutional network to generate a spatial weight map $G\in R^{B\times 1\times H\times W}$, which is normalized via Sigmoid:

(11) $$G=\sigma ({\mathrm{Conv}}_{1\times 1}\left(\mathrm{ReLU}\left({\mathrm{Conv}}_{1\times 1}\left(\left[X,F_{\mathrm{cooccur}}\right]\right)\right)\right)$$

To mitigate background interference, we compute a binary food mask $M_{\mathrm{food}}$ based on the background class probability:

(12) $$M_{\mathrm{food}}=I\left(\mathrm{Softmax}\left(L_{\left[:,0:1\right]}\right)<0.5\right)$$

where I(·) is the indicator function. The final output *Y* is computed via a residual connection:

(13) $$Y=X+\left(G⊙M_{\mathrm{food}}\right)⊙F_{\mathrm{cooccur}}$$

where ⊙ denotes element-wise multiplication. This formulation allows the module to dynamically modulate the contribution of co-occurrence features at each spatial location, ensuring robustness and context-aware enhancement.

Then, the segmentation head consists of a single 1×1 convolutional layer that maps the *C*-dimensional features to $N_{c}$ class logits. The logits are then upsampled to the original resolution via bilinear interpolation and followed by a softmax operation:

(14) $$\mathrm{Logits}={\mathrm{Conv}}_{1\times 1}^{C\to N_{c}}\left(F_{refined}\right)$$

(15) $$P\left(i,j,k\right)=\frac{\exp\left({\mathrm{Upsample}}_{4\times }\left(\mathrm{Logits}\right)\left(i,j,k\right)\right)}{\sum_{k^{\prime }}\exp\left({\mathrm{Upsample}}_{4\times }\left(\mathrm{Logits}\right)\left(i,j,k^{\prime }\right)\right)}$$

The model is trained end-to-end using the standard cross-entropy loss:

(16) $$L_{CE}=-\frac{1}{HW}\sum_{i,j,k}y_{i,j,k}\log P\left(i,j,k\right)$$

### 2.3. Experimental Setup

All experiments were conducted under a unified training and evaluation protocol to ensure fair and reproducible comparison across datasets. The proposed method was implemented using Python 3.12 and PyTorch 2.6.0, and trained on a workstation running Ubuntu 20.04 LTS, equipped with an NVIDIA A800 GPU (80 GB), an Intel^®^ Xeon^®^ Platinum 8358 CPU @ 2.60 GHz, 8 GB RAM, and a 1 TB SSD.

For the FoodSeg103 dataset, the training set consists of 4983 images with corresponding pixel-level masks, while the test set contains 2135 images. All images were resized to 768×768, and a batch size of 2 was used throughout training. For the UEC-FoodPIX Complete dataset, 9000 images were used for training and 1000 images for testing, following the same preprocessing strategy. Images were resized to 768×768, and the batch size was consistently set to 2 to ensure comparable experimental conditions across datasets.

The model was fine-tuned using the official pre-trained weights provided by MambaVision. We employed the AdamW optimizer with an initial learning rate of $6\times 10^{-5}$ and adopted a polynomial learning rate decay strategy (power=1.0). The total number of training iterations was 160,000. The loss function combines cross-entropy loss and Dice loss with a weight ratio of 1:1.

### 2.4. Evaluation Metrics

To comprehensively assess the performance of the proposed HDF framework in food image semantic segmentation, we adopt three widely used evaluation metrics: mean Intersection-over-Union (mIoU), mean Class Accuracy (mAcc), and overall Pixel Accuracy (aAcc). These metrics jointly reflect segmentation quality from region-level overlap, class-wise consistency, and pixel-level prediction accuracy, respectively, which are critical for evaluating fine-grained food segmentation performance in practical applications. Among these metrics, mIoU serves as the primary evaluation criterion, as it directly measures the overlap between predicted segmentation regions and ground-truth annotations across all food categories. This property makes mIoU particularly suitable for food image segmentation tasks, where accurate delineation of ingredient boundaries and balanced performance across diverse food categories are essential. For each class *c*, the Intersection-over-Union (IoU) is defined as

(17) $${\mathrm{IoU}}_{c}=\frac{{\mathrm{TP}}_{c}}{{\mathrm{TP}}_{c}+{\mathrm{FP}}_{c}+{\mathrm{FN}}_{c}}$$

where ${\mathrm{TP}}_{c}$ denotes the True Positive for class *c*, ${\mathrm{FP}}_{c}$ denotes the False Positive, and ${\mathrm{FN}}_{c}$ denotes the False Negative. The mean Intersection-over-Union (mIoU) computes the average of IoU across all classes:

(18) $$\mathrm{mIoU}=\frac{1}{N}\sum_{c=1}^{N}{\mathrm{IoU}}_{c}$$

where *N* is the total number of classes. A higher mIoU value indicates better segmentation performance. The mAcc measures the accuracy for each class individually, avoiding the influence of class imbalance on the evaluation results. For class *c*, the mean class accuracy (mAcc) is the arithmetic mean of all class accuracies:

(19) $$\mathrm{mAcc}=\frac{1}{N}\sum_{c=1}^{N}\frac{{\mathrm{TP}}_{c}}{{\mathrm{TP}}_{c}+{\mathrm{FN}}_{c}}$$

This metric ensures that each class contributes equally to the final evaluation result, effectively reflecting the model’s balanced performance across different food categories. The aAcc directly calculates the proportion of all correctly classified pixels:

(20) $$\mathrm{aAcc}=\frac{\sum_{c=1}^{N}{\mathrm{TP}}_{c}}{\sum_{c=1}^{N}\left({\mathrm{TP}}_{c}+{\mathrm{FN}}_{c}\right)}$$

## 3. Results

### 3.1. Comparative Performance Evaluation

**Figure 5:**
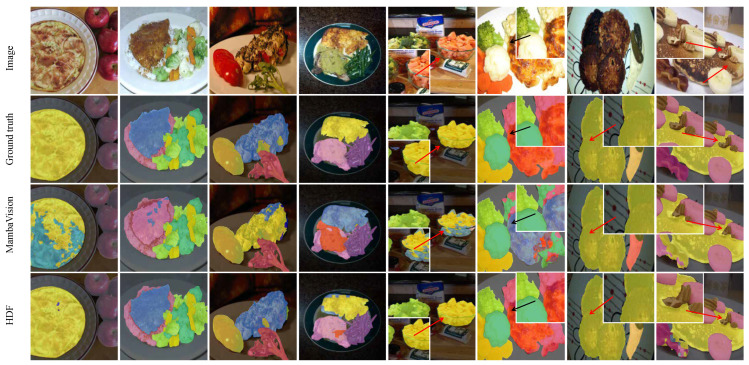
Qualitative comparison of segmentation results on the FoodSeg103 dataset. From top to bottom: input image, ground truth mask, and predictions from different methods (MambaVision, HDF). The proposed HDF method demonstrates superior ability in delineating complex boundaries and distinguishing visually similar ingredients, producing more accurate and coherent segmentation masks.

Table 1 and Figure 5 present a quantitative comparison between the proposed HDF framework and existing state-of-the-art food image segmentation methods on the FoodSeg103 dataset. As shown, HDF achieves an mIoU of 52.25%, representing a 4.91 percentage point improvement over the previous best-performing method, FDSNet [37]. This result demonstrates the effectiveness of the proposed hybrid decoding strategy in addressing the complex visual characteristics of real-world food imagery, including ambiguous boundaries and high inter-class similarity.

**Figure 6:**
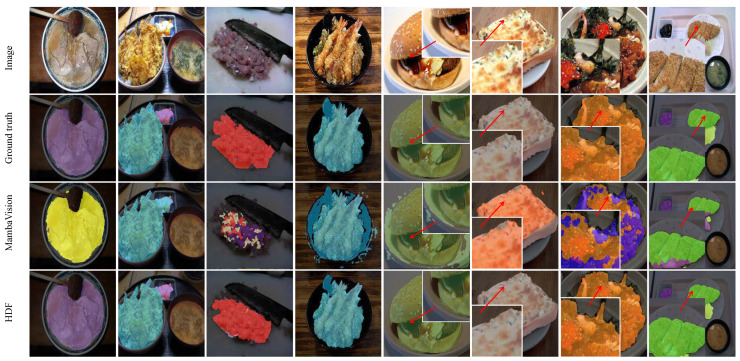
Qualitative segmentation results on the UEC-FoodPIX Complete dataset. The top row shows original Japanese dish images, followed by ground truth masks (second row) and segmentation predictions from MambaVision and the proposed HDF model (third and fourth rows).

To further assess the generalization capability of HDF across diverse food datasets, we conducted additional experiments on the UEC-FoodPIX Complete dataset. The quantitative results, summarized in Table 2 and Figure 6, indicate that HDF attains an mIoU of 76.16%, outperforming competing methods by a clear margin. Notably, the consistent performance gains observed across both datasets suggest that HDF is not over-specialized to a single food domain, but instead provides robust segmentation performance under varying food styles, presentation patterns, and cultural contexts.

### 3.2. Ablation Study and Component Analysis

To comprehensively evaluate the contribution of each component within the proposed HDF architecture, we conducted a systematic ablation study on the FoodSeg103 and UEC-FoodPIX Complete datasets (Table 3 and Table 4). The ablation experiments follow an incremental design, starting from a simple baseline decoder and progressively incorporating each proposed module. All experiments were conducted under identical training settings using the MambaVision Large backbone, an input resolution of 768×768, and a batch size of 2.

The results of the incremental ablation study reveal the distinct and complementary contributions of each architectural component. The introduction of the enhanced feature pyramid network (FPN) yields substantial performance improvements over the single-scale baseline, underscoring the critical importance of multi-scale feature fusion in food image segmentation, where food items often vary significantly in size and spatial extent. Building upon this foundation, the addition of the Cross-Layer Mamba module further improves segmentation accuracy across both datasets. This improvement highlights the effectiveness of modeling inter-layer dependencies to capture complex spatial and contextual relationships inherent in food scenes.

Subsequently, the Multi-Scale Enhancement module refines feature representations at multiple receptive fields, contributing to more accurate delineation of food boundaries, particularly for visually complex or irregularly shaped foods. The Attention Refinement module further enhances segmentation performance by adaptively reweighting spatial and channel-wise features, which is especially beneficial for distinguishing food items with similar textures or colors. Finally, the incorporation of the Food Co-occurrence Module provides an additional performance gain by explicitly modeling common semantic relationships in food scenes, such as the frequent co-occurrence of staple foods with side dishes. This result demonstrates the value of leveraging food-domain knowledge to complement purely visual feature learning.

Overall, the ablation results confirm that each proposed component contributes meaningfully to the final performance of HDF, validating the necessity of the modular design and the synergistic integration of multiple representational mechanisms.

### 3.3. Analysis of the Food Co-Occurrence Module

**Figure 7:**
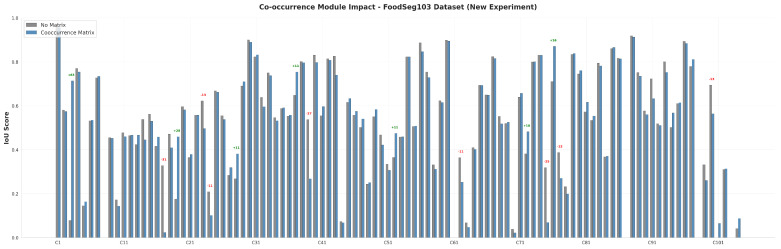
Per-class IoU changes from the co-occurrence module on the FoodSeg103 dataset.

**Figure 8:**
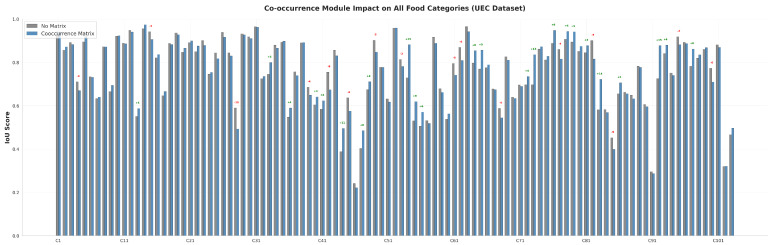
Impact of the co-occurrence matrix on per-class IoU across food categories in the UEC dataset.

To further investigate the behavior of the proposed Food Co-occurrence Module, we conducted a detailed per-class IoU analysis on both benchmark datasets. Figure 7 and Figure 8 illustrate the category-wise IoU changes on FoodSeg103 and UEC-FoodPIX Complete, respectively.

On the FoodSeg103 dataset, the co-occurrence module exhibits substantial performance variations across the 104 food categories. Notable improvements are observed for several underrepresented or visually ambiguous categories, including egg tart (+63.41% IoU, from 7.96% to 71.37%), red beans (+28.33% IoU), and date (+3.41% IoU). These gains indicate that co-occurrence modeling can effectively compensate for limited visual cues by leveraging contextual relationships. Conversely, performance degradations are observed for certain single-ingredient or visually isolated categories, such as tea (−30.5% IoU, from 32.88% to 2.38%), pear (−27% IoU), and walnut (−12.58% IoU).

On the UEC-FoodPIX Complete dataset, the co-occurrence module demonstrates more stable but relatively modest improvements. Specifically, 68 out of 103 categories (66.0%) exhibit IoU gains, with the most significant improvements observed for steamed egg hotchpotch (+15.32% IoU), Chinese soup (+15.21% IoU), and spicy chili-flavored tofu (+8.68% IoU). In contrast, mixed rice experiences the largest performance degradation (−6.32% IoU).

These results reveal several important patterns. First, complex dishes containing multiple visible ingredients tend to benefit more from co-occurrence modeling than single-ingredient categories, highlighting the contextual nature of food scene understanding. Second, the differing performance trends across datasets suggest sensitivity to dataset composition, cultural food practices, and annotation characteristics. Finally, the presence of both improvements and degradations indicates that while co-occurrence modeling is beneficial, its effectiveness is influenced by training data distribution and category semantics. These observations point to important directions for further refinement of context-aware food segmentation models.

## 4. Discussion

The proposed HDF framework achieves state-of-the-art performance on fine-grained food image segmentation by synergistically integrating three complementary representation paradigms: CNNs for local detail fidelity, Transformers for global semantic reasoning, and Mamba for efficient long-range dependency modeling. This hybrid design directly addresses the core challenges outlined in the Introduction, including ambiguous boundaries, high inter-class similarity, and dense layouts of dishes with multiple ingredients commonly encountered in real-world food scenes. A key innovation is the FCM, which enhances segmentation accuracy for complex multi-component dishes by explicitly learning semantic and spatial relationships between food categories. This demonstrates the value of incorporating structured data driven domain knowledge to complement visual feature learning.

A comparative analysis with two notable contemporary approaches, FoodSAM [22] and FDSNet [37], further contextualizes the contributions and limitations of HDF. In terms of semantic segmentation accuracy, HDF outperforms FoodSAM, which is built upon the SAM [21]. For instance, HDF achieves a 52.25% mIoU compared to FoodSAM’s 46.42% on the FoodSeg103 dataset. This advantage stems from HDF’s dedicated architecture, which is specifically optimized for the nuances of fine-grained food parsing rather than relying on a general-purpose foundation model. However, FoodSAM represents a complementary paradigm with distinct strengths, notably its remarkable zero-shot generalization capability and support for a broader range of tasks, including instance, panoptic, and promptable segmentation, within a unified framework. This functional versatility is not a focus of the current HDF design.

When compared with FDSNet, which employs an efficient dual-branch architecture, HDF achieves higher segmentation accuracy. However, this performance gain comes with a significant increase in computational cost. The HDF decoder introduces approximately 28.9 million parameters and requires around 621 GFLOPs. In contrast, FDSNet maintains competitive accuracy with only 101.93 million parameters and 182.74 GFLOPs, by processing high-resolution details through a lightweight shallow branch and downsampled images through a deep branch. HDF’s pursuit of optimal accuracy through a complex sequential decoder design elevates its computational demands, which could hinder its real-time deployment in resource-constrained environments such as mobile devices for dietary assessment.

Furthermore, although the FCM’s dynamic adjustment mechanism offers an advantage over static co-occurrence priors and benefits many categories, we observed a slight performance dip for some frequently appearing food items. This suggests that the adaptive gating mechanism may not always allocate optimal weights for dominant categories during feature fusion, potentially diluting their strong visual signatures. Future refinements could explore normalization strategies that account for category frequency or more calibrated weighting within the FCM to ensure balanced improvement across both rare and common categories.

To address these challenges, future research will focus on several promising directions. Architecturally, exploring lightweight decoder designs, module pruning, or knowledge distillation techniques could dramatically improve efficiency while preserving accuracy. To mitigate data bias and improve generalization, future work could investigate curriculum learning that emphasizes rare categories, develop co-occurrence modeling that adapts to cultural context, and integrate multimodal information such as textual recipes or depth cues to provide complementary signals. Finally, extending the hybrid encoder–decoder framework to support instance-aware or prompt-driven segmentation would broaden its applicability, potentially by integrating lightweight prompting mechanisms or designing auxiliary prediction heads. By advancing along these complementary paths, the goal of building accurate, efficient, robust, and versatile food image segmentation systems for nutrition applications becomes more attainable.

## 5. Conclusions

This study presents HDF, a hybrid decoding framework designed to address key challenges in fine-grained food image segmentation, including multi-scale object variability, complex visual textures, and ambiguous boundaries commonly encountered in real-world food scenes. By integrating an enhanced feature pyramid network, Cross-Layer Mamba modeling, multi-scale feature enhancement, Attention Refinement, and a spatially aware Food Co-occurrence Module, HDF effectively combines local detail preservation with global contextual reasoning while maintaining computational efficiency. Comprehensive experiments on the FoodSeg103 and UEC-FoodPIX Complete datasets demonstrate that HDF consistently outperforms existing state-of-the-art methods. Ablation studies further validate the complementary contributions of each architectural component, highlighting the effectiveness of the proposed modular design in capturing both visual and semantic relationships among food items. In particular, the explicit modeling of food co-occurrence patterns proves beneficial for complex, multi-ingredient dishes, underscoring the value of incorporating food domain knowledge into segmentation pipelines. From an application perspective, the proposed framework provides a robust foundation for practical food computing systems, including mobile nutritional assessment, automated dietary logging, and food-safety-related analysis. At the same time, the observed limitations related to computational cost, data imbalance, and cultural specificity point to important directions for future research.

## Figures and Tables

**Table 1:** Comparison of HDF with state-of-the-art methods on the FoodSeg103 dataset across standard segmentation metrics.

Methods	mIoU (%)	mAcc (%)	aAcc (%)
DeeplabV3+ [38]	36.22	48.87	–
STPPN [39]	40.30	53.98	82.13
FoodSAM [22]	46.42	58.27	84.10
UperNet [32]	39.80	52.37	82.02
FDSNet (Swin) [37]	47.34	60.04	–
MambaVision [31]	49.95	62.29	85.34
HDF (Ours)	**52.25**	**64.78**	**85.99**

**Table 2:** Performance comparison of HDF and representative baselines on the UEC-FoodPIX Complete dataset.

Methods	mIoU (%)	mAcc (%)	aAcc (%)
CANet [40]	68.90	80.60	–
DeepLabV3+ [38]	65.61	77.56	88.20
GourmetNet [41]	62.88	75.87	87.07
FoodSAM [22]	66.14	78.01	88.47
BayesianDeepLabv3+ [42]	64.21	76.15	87.29
PSPNet (Fine-tuned on Food2K)	74.50	84.10	–
FDSNet (Swin) [37]	75.89	86.29	–
MambaVision [31]	75.01	86.09	91.07
HDF (Ours)	**76.16**	**86.83**	**91.63**

**Table 3:** Ablation study on FoodSeg103 showing the contribution of each proposed component in HDF.

Components	mIoU (%)	mAcc (%)	aAcc (%)
Baseline (FCNHead)	44.51	58.35	84.16
+FPN	48.89	63.91	84.98
+Cross-Layer Mamba	49.91	64.25	85.59
+Multi-Scale Enhancement	50.15	64.74	85.51
+Attention Refinement	51.66	64.47	86.31
+Food Co-occurrence Module (FCM)	**52.25**	**64.78**	**85.99**

**Table 4:** Ablation study on UEC-FoodPIX Complete evaluating the incremental impact of HDF’s modules.

Components	mIoU (%)	mAcc (%)	aAcc (%)
Baseline (FCNHead)	73.09	85.90	90.03
+FPN	73.43	86.00	90.20
+Cross-Layer Mamba	74.01	85.30	90.46
+Multi-Scale Enhancement	74.49	85.41	90.50
+Attention Refinement	75.45	85.66	91.31
+Food Co-occurrence Module (FCM)	**76.16**	**86.83**	**91.63**

## Data Availability

The original contributions presented in this study are included in the article. Further inquiries can be directed to the corresponding author.

## Appendix A

This appendix provides the complete architectural details, formulations, and diagrams for the Hybrid Encoder described in Section 2.2.1. It includes the specifications for the downsampling stem, the residual block designs, the structure of the MambaVision Mixer, and the State Space Model:

(A1) $$X_{0}={\mathrm{Conv}}_{3\times 3}^{\mathrm{stride}=2}\left(I\right)\in R^{H/2\times W/2\times C}$$

(A2) $$X_{\mathrm{stem}}={\mathrm{Conv}}_{3\times 3}^{\mathrm{stride}=2}\left(X_{0}\right)\in R^{H/4\times W/4\times C}$$

(A3) $$\hat{X}=X+{\mathrm{Conv}}_{3\times 3}\left(\mathrm{GELU}\left(\mathrm{BN}\left({\mathrm{Conv}}_{3\times 3}\left(X\right)\right)\right)\right)$$

(A4) $${\hat{X}}_{n}=\mathrm{Mixer}\left(\mathrm{LN}\left(X_{n-1}\right)\right)+X_{n-1}$$

(A5) $$X_{n}=\mathrm{MLP}\left(\mathrm{LN}\left({\hat{X}}_{n}\right)\right)+{\hat{X}}_{n}$$

(A6) $$X_{\mathrm{out}}=\hat{X}+\mathrm{MLP}\left(\mathrm{GELU}\left(\mathrm{BN}\left(\hat{X}\right)\right)\right)$$

(A7) $$X_{1}=\mathrm{Scan}\left(\sigma \left(\mathrm{Conv}\left({\mathrm{Linear}}^{C\to C/2}\left(X_{in}\right)\right)\right)\right)$$

(A8) $$X_{2}=\sigma \left(\mathrm{Conv}\left({\mathrm{Linear}}^{C\to C/2}\left(X_{in}\right)\right)\right)$$

(A9) $$X_{out}={\mathrm{Linear}}^{C/2\to C}\left(\left[X_{1};X_{2}\right]\right)$$

(A10) $$h\left(t\right)=\tilde{A}h\left(t-1\right)+\tilde{B}x\left(t\right)$$

(A11) $$y\left(t\right)=\tilde{C}h\left(t\right)$$

(A12) $$\mathrm{Attention}\left(Q,K,V\right)=\mathrm{Softmax}\left(\frac{QK^{T}}{\sqrt{d_{h}}}\right)V$$
